# Peripheral perfusion and acute mountain sickness: is there a link?

**DOI:** 10.1186/2046-7648-4-S1-A46

**Published:** 2015-09-14

**Authors:** Adam C McDonnell, Ola Eiken, Igor B Mekjavic

**Affiliations:** 1 Department of Automation, Biocybernetics and Robotics, Jozef Stefan Institute, Jamova Cesta 39, 1000 Ljubljana, Slovenia; 2 Jozef Stefan International Postgraduate School, Jamova Cesta 39, 1000 Ljubljana, Slovenia; 3 Department of Environmental Physiology, Swedish Aerospace Physiology Centre, Royal Institute of Technology, Stockholm, Sweden

## Introduction

It has been suggested that there is an interaction between acute mountain sickness, body temperatures and autonomic responses [1]. We tested this hypothesis by monitoring the circadian rhythm of peripheral perfusion reflected in the proximal to distal skin temperature gradient (ΔTp-d) during acute exposure of active and inactive (bedrest experimental protocol) subjects to acute normobaric hypoxia [2].

## Methods

Thirty-six males participated in a total of 58 hypoxic exposures within the framework of three separate studies: LunHab (11 participants; two 10 day hypoxic confinements: P_I_O_2 _= 103 mmHg, simulated altitude of 3,000 m), PlanHab, (11 participants; two 21 day hypoxic confinements: P_I_O_2 _= 89.6 mmHg, simulated altitude of 4,000 m), HECS (14 participants; one 10 day hypoxic confinement: P_I_O_2 _= 88.2 mmHg, simulated altitude of 4,175 m). On the first day of exposure to the simulated hypoxic condition, all participants of each study entered the hypoxic area at 9:00. Proximal to distal skin temperature gradient (calf and toe respectively, ΔTc-t) was recorded continuously from 10:00 for 20 hrs on this first day of exposure (D1). The participants completed the Lake Louise Mountain Sickness (LLMS) question in the evening of the first day between 20:00 and 21:00. Distal and proximal skin temperatures were recorded at 1-minute intervals and then averaged into 60-minute epochs. The ΔTc-t data was collated into two groups, those who presented with acute mountain sickness (AMS) and those who did not (nAMS).

## Results

Of the 58 exposures, sixteen resulted in participants presenting with mild symptoms of AMS on D1 of the hypoxic exposure (4.2 (1.3)). The nAMS group (42 exposures) gave an average rating of 0.4 (0.7) on the LLMS questionnaire. The ΔTc-t measured in the AMS group increased from 4.1 (3.4) °C at 17:00 to 5.1 (3.7) °C at 18:00 and to 5.0 (3.7) °C at 19:00. The nAMS group had a ΔTc-t of 2.6 (2.9) °C at 18:00 and 3.1 (3.1) °C at 19:00. A significant difference (p < 0.05) in the ΔTc-t gradient between the AMS and nAMS group persisted until 19:00 on D1 of the hypoxic exposure. A non-significant difference (p = 0.06) was observed between 19:00 and 20:00. At 21:00 the difference in ΔTc-t between the two groups had abated, whereby both groups showed significant vasodilatation (p < 0.05) of the toes thereafter and throughout the night compared to the daytime. There were no differences between groups in terms of age, body mass, body fat % or VO_2max_.

## Conclusions

The main finding of the current study is that daytime vasoconstriction of the periphery seen during hypoxic confinement is more pronounced in those suffering from AMS during the evening hours (17:00 to 19:00). This increased hypoxia-induced vasoconstriction in individuals with AMS suggests that they may be at a greater risk of cold injury when exposed to extreme cold at high altitude. Acknowledgements: The research leading to these results has received funding from the European Union's Framework Programme (2007-2013) under grant agreement no. 284438 (project PlanHab: Planetary Habitat Simulation), the European Space Agency (ESA) Programme for European Cooperating States (ESTEC/Contract No. 40001043721/11 / NL / KML: Planetary Habitat Simulation) and the Slovene Research Agency (Project L3-4328).

## References

[B1] Loeppky JA Icenogle MV Maes D Riboni K Scotto P Roach RC Body temperature, autonomic responses, and acute mountain sickness High Alt Med Biol 2003 4 3 367 373 10.1089/152702903769192322 14561242

[B2] McDonnell AC Eiken O Mekjavic PJ Mekjavic IB Circadian rhythm of peripheral perfusion during 10-day hypoxic confinement and bed rest Eur J Appl Physiol 2014 114 10 2093 2104 10.1007/s00421-014-2923-9 24943734

